# The Role of Nurse Leadership in the Implementation of Evidence-Based Practices in Oncology Care: A Focus on Spain

**DOI:** 10.7759/cureus.84858

**Published:** 2025-05-26

**Authors:** Chidinma S Madu, Victoria M Ajibade

**Affiliations:** 1 Neuro-Oncology, Duke University Health System, Durham, USA; 2 Pediatric Critical Care, Duke University Health System, Durham, USA

**Keywords:** clinical research, evidence-based practice, interdisciplinary collaboration, nurse leadership, oncology nursing, patient outcomes, quality care

## Abstract

Oncology nursing is a dynamic and evolving field that requires high-quality, research-informed care to meet the complex needs of cancer patients. The integration of evidence-based practices (EBPs) is crucial for improving clinical outcomes, enhancing symptom management, and promoting patient-centered care. Nurse leaders play a pivotal role in advancing EBP adoption by demonstrating transformational leadership, providing mentorship, and advocating for supportive organizational structures. Professional nursing also demands active engagement in research, both as contributors to the evidence base and as informed consumers of research findings. Despite this, barriers such as resistance to change, limited time, and insufficient institutional support often hinder the effective implementation of EBPs. Overcoming these challenges through increased research funding, interdisciplinary collaboration, and the use of innovative technologies is key to cultivating a culture of evidence-based oncology nursing. Strong nurse leadership remains essential in bridging the gap between research and clinical practice, ultimately ensuring the delivery of high-quality oncology care.

## Editorial

Oncology nursing care is undergoing significant changes on an international scale. Clinical practices are evolving in response to advances in technology, economic constraints, shorter hospital stays, an aging population, and increased patient involvement in decision-making. Nurses remain the largest group of healthcare professionals and deliver the majority of patient care services, making their role in healthcare delivery essential. In Spain, these global trends are equally relevant. Nurses are expected to meet these challenges with professional excellence, often within the limits of constrained resources [1].

In the context of healthcare expenditure, there is a growing shift from focusing on procedures to evaluating care outcomes. This change has driven an increase in rigorous and time-intensive nursing research and has promoted the integration of evidence-based practices (EBPs) aimed at delivering more effective and efficient care.

Professional nursing organizations in Spain play a vital role in fostering and supporting nursing research to improve healthcare outcomes. For instance, the Official College of Nursing of Barcelona grants annual awards to recognize excellence in nursing research. Meanwhile, cancer mortality in Spain has declined markedly in recent years. A comparative study of cancer mortality rates across European Union (EU) countries during the 1990-1994 period ranked Spain ninth for men and 12th for women. The reduction in mortality was particularly notable among women, positioning Spain among the EU countries with the lowest cancer mortality rates [2].

This growing population of cancer survivors reflects improved access to advanced medical technologies and enhanced professional standards in care. Nevertheless, there continues to be debate about whether progress in clinical practice and knowledge can be achieved solely through high-quality research and education. In Spain, nursing education was incorporated into the university system 25 years ago, initially as a diploma program. Notably, Spain was the first EU country to grant full university-level recognition to nursing education.

Since then, Spanish nursing has advanced significantly, now offering bachelor’s, master’s, and doctoral degree programs. Other EU countries, including Portugal, the Netherlands, Italy, Finland, Greece, Ireland, England, and Iceland, have also adopted similar academic frameworks.

The Spanish Ministry of Education, by signing the Bologna Agreement, committed to aligning university education with the three-tier academic structure of bachelor’s, master’s, and doctoral degrees by 2010. As a result, Spanish nursing education is required to follow this framework. To ensure the ability to conduct high-quality research, Spanish nurses must pursue advanced education through master’s and doctoral programs. Doctoral education remains the primary pathway for acquiring rigorous research training, which can be tailored to specialized fields such as oncology nursing. Bridging the gap between theoretical knowledge and clinical practice requires a combination of academic preparation and professional experience.

Globally, advanced nursing education has significantly contributed to building expertise in various areas of cancer care, including managing chemotherapy side effects, supporting patient adaptation, enhancing quality of life, promoting social support, delivering home care, and advancing cancer prevention and early detection. This specialized training has also opened doors to leadership opportunities and active involvement in decision-making processes.

These advancements support the case for reallocating research funding and prioritizing key areas in cancer nursing. Countries with well-established doctoral nursing programs have seen exponential growth in awards recognizing outstanding research. In Spain, institutions such as the San Juan de Dios School of Nursing and Physiotherapy (Comillas Pontifical University-Juan Ciudad Foundation) in Madrid, along with other official colleges of nursing, acknowledge and promote excellence in nursing research. The Spanish Oncology Nursing Association (AEEO) also plays a vital role in promoting research through its publications and conferences. Representing over 400 oncology nurses across Spain, the AEEO includes professionals working in hospitals, primary care, and home care settings. One of its core goals is to initiate and support research aimed at improving cancer patient care and outcomes.

Historically, the integration of empirical evidence into clinical practice has posed a challenge for healthcare professionals. Despite the findings that were demonstrated on research-based interventions that produce better outcomes than routine care, many nurses still rely on knowledge gained through diploma-level education, personal experience, or peer advice. This reliance has led to considerable variability in clinical practice - even among nurses working in the same unit - often without full awareness of the latest scientific developments [3].

To strengthen research capacity in oncology nursing, professional associations should establish expert research panels to provide strategic guidance. Key objectives for these panels should include (a) disseminating relevant research findings; (b) offering guidance on specific research topics; (c) identifying priority research areas within Spanish oncology nursing; and (d) developing research networks focused on key projects and themes.

Despite the global push toward higher academic training and EBP, a persistent gap remains between theoretical knowledge, research evidence, and its practical application in clinical nursing interventions [4]. To improve cancer patient survival rates and quality of life, the nursing profession in Spain must focus on four critical areas:

Securing increased research funding

A major barrier to nursing research is insufficient funding. Historically, the nursing profession has faced challenges in obtaining financial support for research, resulting in a limited but valuable body of evidence. Doctoral training enhances the quality of clinical care when findings are applied in practice. Nurses can begin accessing funding at the local level through university or hospital grants, which can then serve as a foundation for applying for national or international funding. Nursing leaders must also advocate for financial support from pharmaceutical companies to promote oncology nursing research. Each year, the Carlos III Health Institute, through the Health Research Fund (FIS), provides funding for research on clinical nursing interventions. Given the complexity of clinical decision-making and patient care, FIS prioritizes multidisciplinary research projects, offering valuable opportunities for oncology nursing research.

Enhancing nursing research productivity

In addition to funding challenges, limited research output is also linked to a lack of training and a shortage of studies on clinically relevant topics. Many existing studies, characterized by small sample sizes and limited scientific rigor, have shown that training is positively associated with nurses’ confidence in engaging in research, underlining the importance of continued education to foster EBP. Doctoral programs allow nurses to collaborate with seasoned researchers, apply for funding, conduct robust research, and publish their findings. This not only strengthens individual research competencies and enhances professional credibility but also improves access to funding opportunities covering research costs, clinical leave, and consultancy fees. Ultimately, this contributes to a stronger scientific foundation for nursing practice and elevates the profession’s visibility in both medical and academic spheres. Patients and communities benefit as well, receiving care grounded in evidence rather than tradition, leading to better outcomes and potentially more efficient healthcare delivery.

Improving dissemination of research findings

One of the enduring challenges in nursing research is the effective dissemination and implementation of findings into clinical practice. Identifying optimal dissemination strategies and user-friendly publication platforms is essential. The AEEO publishes Oncology Nursing, a professional journal dedicated to cancer care, along with several other journals that occasionally feature oncology-related studies. Spanish nurses are increasingly incorporating scientific literature into their practice, although international benchmarks suggest room for improvement. For example, in Australia, 82% of nurses report regularly reading literature in their field to stay updated. While reading research alone does not guarantee changes in clinical practice, it is a crucial first step toward achieving evidence-based care.

Promoting the practical application of research findings

To ensure that nursing research has a tangible impact on patient care, it is essential to focus on the practical application of research findings. This involves translating evidence into clinical protocols, decision-making tools, and day-to-day nursing practices. In the context of Spanish oncology nursing, fostering stronger collaboration between researchers, clinical nurses, and healthcare administrators can bridge the gap between research and practice. Continuing education programs, implementation science initiatives, and institutional support for EBP are crucial to embedding research outcomes into clinical workflows. By promoting the practical application of research, the profession can ensure that scientific knowledge directly benefits patients, enhances care quality, and supports innovation in oncology settings.

By addressing these four key areas - research funding, productivity, dissemination, and practical application - Spanish oncology nursing can continue progressing toward full integration of scientific evidence into practice. This will ultimately lead to better patient outcomes and higher-quality cancer care.

Oncology nursing is a dynamic and rapidly evolving discipline that demands practitioners stay at the forefront of research advancements, clinical innovations, and best practices to deliver optimal patient care. The increasing complexity of cancer treatment, combined with continual advancements in therapeutic modalities, underscores the importance of oncology nurses remaining current with the latest EBPs. Doing so supports improved patient outcomes, more effective symptom management, and greater overall patient satisfaction [5]. EBPs not only elevate the quality of care but also promote standardized treatment approaches, reducing variability in clinical decision-making and ensuring consistency in patient management.

In this ever-changing landscape, nurse leaders play a critical role in bridging the gap between research and clinical practice. These leaders, including nurse managers, advanced practice nurses, and clinical nurse specialists, are key facilitators in cultivating environments that support the integration of EBPs. Their influence extends beyond bedside care, encompassing institutional policy development, the promotion of continuous learning, and the mentorship of frontline nurses in applying evidence-based interventions [6]. By spearheading the implementation of research-driven strategies, nurse leaders help ensure oncology nursing remains progressive, adaptable, and aligned with the latest clinical advances.

To effectively integrate EBPs within oncology settings, several leadership strategies have proven essential. Transformational leadership, which focuses on inspiration, motivation, and professional growth, has been shown to significantly support EBP adoption. Transformational nurse leaders empower their teams by creating a shared vision, encouraging innovation, and providing the resources and support needed to overcome resistance and implement change. Mentorship programs also play a vital role in guiding less experienced nurses through the complexities of evidence-based oncology care [7]. By pairing novice nurses with experienced mentors, healthcare organizations can build a confident workforce that utilizes research findings to inform clinical decisions.

In addition to mentorship, nurse leaders actively engage in policy advocacy to build organizational structures that prioritize research and the application of EBPs [8]. This advocacy includes efforts to secure increased funding for nursing research, protect time for nurses to engage in scholarly activities, and collaborate with interdisciplinary teams to streamline the implementation of new evidence-based protocols. By influencing both institutional and broader policy decisions that support ongoing education and research, nurse leaders help establish a sustainable framework for delivering high-quality, evidence-informed oncology care [9].

Despite the well-documented benefits of EBPs, several barriers to their implementation remain. Common challenges include resistance to change, time limitations, restricted access to research literature, and insufficient institutional support. Addressing these obstacles requires a multifaceted strategy that combines continuous professional development, the strategic use of technology for knowledge dissemination, and a collaborative, interdisciplinary approach to care. Nurse leaders must take an active role in overcoming these challenges by advocating for access to professional development opportunities, facilitating the use of research databases, and integrating digital tools into everyday clinical workflows to support seamless EBP implementation [10].

Ultimately, the evolving nature of oncology nursing highlights the vital importance of strong nurse leadership in advancing EBP. By championing research-informed care models, employing effective leadership strategies, and addressing systemic barriers to EBP adoption, nurse leaders play a pivotal role in ensuring oncology patients receive the highest standard of care [11]. Their efforts not only shape the current and future landscape of oncology nursing but also drive continuous improvements in patient outcomes, symptom control, and overall healthcare efficiency.

Challenges and future directions

Despite the well-documented benefits of EBPs in oncology nursing, several obstacles hinder their widespread adoption. One of the primary challenges is resistance to change among nursing staff. Some nurses may be reluctant to alter established routines or skeptical about how research findings apply in real-world clinical settings. This hesitation is often worsened by low staff engagement, where nurses feel disconnected from institutional efforts to implement EBPs, resulting in reduced motivation to incorporate research-driven interventions into their practice [12].

Limited access to research resources and insufficient training also present significant barriers [13]. Many oncology nurses struggle to obtain the latest research due to restricted access to scientific journals, limited institutional support for professional development, and inadequate training in research appraisal and implementation. Without proper education and easy access to current research, nurses face challenges integrating EBPs effectively into patient care.

Institutional constraints further impede EBP adoption. Insufficient funding for research initiatives and staffing shortages make it difficult for nurses to dedicate time to learning about and applying EBPs [14]. High patient loads and administrative duties reduce opportunities for nurses to engage in research-related activities, perpetuating the gap between evidence and practice [15].

To overcome these challenges, future efforts should emphasize leadership development programs that equip nurse leaders with the skills to drive EBP integration. Increased funding for research and the use of technology, such as digital decision-support tools, can provide nurses with real-time access to research-based guidelines. Additionally, strengthening interdisciplinary collaboration and implementing structured mentorship programs offer essential guidance and support, helping nurses adopt EBPs more confidently and improving oncology patient care.

This article provides a comprehensive overview of the pivotal role nurse leaders play in integrating EBPs within oncology care. It highlights how nurse leaders uniquely bridge clinical research and bedside care, ensuring patient treatment plans are grounded in the latest scientific evidence. The authors clearly outline strategies such as fostering a culture of inquiry, mentoring clinical staff, and promoting cross-disciplinary collaboration. Especially noteworthy is the emphasis on empowering nurses through education and leadership development, which directly improves patient outcomes and care quality in oncology settings.

While rich in practical insights, the article could be strengthened by including real-world case studies that demonstrate successful EBP implementation. Nonetheless, it serves as a valuable resource for nursing professionals and healthcare administrators striving to elevate oncology care through strong, evidence-driven leadership.

Conclusions

The future of oncology nursing depends on the seamless integration of EBP, effective leadership, active research engagement, and interprofessional collaboration. As cancer treatments become increasingly complex, oncology nurses must remain flexible to accommodate new modalities and evolving patient needs and deliver high-quality, patient-centered care. This evolution relies on a workforce equipped with up-to-date scientific knowledge, clinical expertise, and the ability to translate research into practice. However, various barriers limit the extent of evidence-based oncology nursing implementation. Resistance among individuals, lack of time for research activities, and insufficient institutional support remain the most significant challenges. Without a solid research foundation, many nurses continue relying on traditional practices rather than EBPs, leading to inconsistencies in care and missed opportunities for improving patient outcomes.

Overcoming these challenges requires healthcare organizations to foster a culture of research that prioritizes continuous learning, professional development, and structured mentorship. Key drivers of progress include advancing education, securing stable research funding, and leveraging technological innovations. Investing in postgraduate training programs, funding nurse-led research projects, and improving access to clinical resources will enhance the profession’s ability to deliver high-quality oncology care. Moreover, interdisciplinary collaboration among nurses, physicians, researchers, and policymakers is essential to expanding the oncology nurse’s role in shaping clinical guidelines and advocating for systemic improvements in cancer care. Ultimately, the future of oncology nursing rests with nurse leaders committed to innovation, policy reform, and creating environments where scientific inquiry and clinical excellence coexist. By leading these efforts, oncology nurses can advance their role in cancer care, ensuring better outcomes and improved quality of life for patients and their families.
